# Anemia is an independent risk for mortality after acute myocardial infarction in patients with and without diabetes

**DOI:** 10.1186/1475-2840-5-8

**Published:** 2006-04-07

**Authors:** David H Shu, Thomas PP Ransom, Colleen M O'Connell, Jafna L Cox, Stephanie M Kaiser, Shirl A Gee, Richard C Rowe, Ehud Ur, Syed Ali Imran

**Affiliations:** 1 Department of Medicine, Dalhousie University, Halifax, Canada; 2 Perinatal Epidemiology Research Unit, Izaak Walton Killam Health Centre, Halifax, Canada; 3 Department of Community Health and Epidemiology, Dalhousie University, Halifax, Canada

## Abstract

**Introduction:**

Anemia and diabetes are risk factors for short-term mortality following an acute myocardial infarction(AMI). Anemia is more prevalent in patients with diabetes. We performed a retrospective study to assess the impact of the combination of diabetes and anemia on post-myocardial infarction outcomes.

**Methods:**

Data relating to all consecutive patients hospitalized with AMI was obtained from a population-based disease-specific registry. Patients were divided into 4 groups: diabetes and anemia (group A, n = 716), diabetes and no anemia (group B, n = 1894), no diabetes and anemia (group C, n = 869), and no diabetes and no anemia (group D, n = 3987). Mortality at 30 days and 31 days to 36 months were the main outcome measures.

**Results:**

30-day mortality was 32.3% in group A, 16.1% in group B, 21.5% in group C, 6.6% in group D (all p < 0.001). 31-day to 36-month mortality was 47.6% in group A, 20.8% in group B, 34.3% in group C, and 10.4% in group D (all p < 0.001). Diabetes and anemia remained independent risk factors for mortality with odds ratios of 1.61 (1.41–1.85, p < 0.001) and 1.59 (1.38–1.85, p < 0.001) respectively at 36 months. Cardiovascular death from 31-days to 36-months was 43.7% of deaths in group A, 54.1% in group B, 47.0% in group C, 50.8% group D (A vs B, p < 0.05).

**Interpretation:**

Patients with both diabetes and anemia have a significantly higher mortality than those with either diabetes or anemia alone. Cardiovascular death remained the most likely cause of mortality in all groups.

## Introduction

Both anemia and diabetes mellitus (DM) are recognized as strong, independent risk factors for mortality and recurrent ischemia after acute myocardial infarction (AMI). [1-6] Anemia is 2–3 times more common in individuals with DM compared to those without DM, controlling for glomerular filtration rates and iron stores[7]. Recent studies have shown that anemia is associated with increased short-term mortality in patients with IHD. However, little is known concerning the long-term survival of IHD patients with anemia. One study suggested no difference in long-term mortality with anemia once various confounding conditions were adjusted for[8]. Since anemia is often a marker of an underlying disease, the actual causes of death associated with a low hemoglobin level are unknown. These patients may be dying of an associated condition and not from cardiovascular disease. Furthermore, the outcome of acute MI patients that have both anemia and diabetes has not been studied to date.

We undertook a study to assess the impact of anemia on both short, and long-term morbidity and mortality in post-AMI patients with and without DM, and to identify the cause-specific mortality in this population.

## Methods

### Study setting, design and population

The Improving Cardiovascular Outcomes in Nova Scotia (ICONS) Study, was a large prospective cohort, disease management initiative whose rationale and methods have been comprehensively described elsewhere[9]. As part of this study, detailed clinical data were collected through primary chart audit by trained abstractors at adult care hospitals throughout the province of Nova Scotia, Canada on all patients with AMI admitted since October 15, 1997 until December 31, 2001. Patients were divided into four groups based on whether or not they had anemia or diabetes on admission. Diabetes was defined as either a previous history of diabetes or admission glucose of > 200 mg/dL based upon the 2003 Canadian Diabetes Association clinical practice guidelines[10]. Anemia was defined as hemoglobin < 12.0 g/dL in females and < 13.0 g/dL in males, according to the World Health Organization criteria for anemia[11].

### Data elements

The data abstracted for analysis included patient demographics and clinical characteristics such as age, sex, body mass index (BMI), blood pressure, type of AMI, smoking status, family history of coronary heart disease, history of previous AMI or previous congestive heart failure (CHF), therapies during hospitalization, cardiac drugs on discharge, mortality, and readmission with either re-infarction, unstable angina, CHF or AMI. Laboratory results that were collected included mean glucose on admission, hemoglobin on admission, lipid profile, serum creatinine kinase, and serum creatinine. Creatinine clearance was calculated using the Cockroft-Gault equation. Individual cause-specific mortality was obtained from the Vital Statistics Registry of the Province of Nova Scotia via the Department of Health, as reported through ICD-9 and ICD-10 codes.

### Outcomes of interest

These included mortality at 30 days and out to 36 months; readmission for unstable angina, or nonfatal myocardial infarction, or congestive heart failure; a composite of all three morbidity endpoints; and cause of death at 30 days and out to 36 months.

### Data analysis

Demographic and clinical characteristics of the study groups were compared using ANOVA for continuous variables and chi-square tests for categorical variables. Those data elements found to differ among the study groups in univariate analyses at a significance level of p < 0.05 were entered into a Cox Proportional Hazards regression model with mortality (either within 30 days or between 31 days and 36 months of index admission) as the measure of interest. A backward elimination method was chosen wherein all potential predictors were entered as a block and removed in a stepwise fashion depending on the relationship of each with the outcome, resulting in the most parsimonious models representing the relationships between the predictor and the outcome variables. For illustrative purposes, patients were categorized into quintiles of hemoglobin levels separately for each sex and a survival curve to 36 months was plotted. All analyses were conducted using SPSS 10.0 (Chicago, IL).

### Ethics review

The original ICONS Study protocol underwent ethics review at the Queen Elizabeth II Health Sciences Centre in Halifax and at participating institutions across Nova Scotia. The present study protocol underwent additional ethics appraisal at the Queen Elizabeth II Health Sciences Centre in Halifax.

## Results

Between October 15, 1997 and December 31, 2001, 7507 patients were admitted with acute MI. Due to incomplete information, 41 patients were excluded from the subsequent analysis. The remaining 7466 patients (99.5%) were included in the study and followed for up to 36 months from their index admission. Patients were divided into four groups on the basis of anemia and diabetes status: group A (patients with DM and anemia [n = 716]), group B (patients with DM but without anemia [n = 1894]), group C (patients without DM but with anemia [n = 869]), and group D (patients without DM and without anemia [n = 3987]).

### Baseline characteristics

Table 1 describes the baseline characteristics of the study sample. The prevalence of anemia in DM patients was 27.4% compared with 17.9% in the non-DM group (p < 0.001). Patients with anemia tended to be older in both the diabetes (74.0 y vs. 67.8 y, p < 0.05) and non-diabetes (76.1 y vs. 64.2 y, p < 0.05) groups. Patients with anemia had lower creatinine clearances (group A = 39.2 mL/min, group B = 61.4 mL/min, group C = 41.5 mL/min, group D = 64.6 mL/min, all p < 0.001, except A and C p=ns). Most patients with anemia had sustained a non-ST elevation MI on admission (Group A = 72.7% and group C = 67.7%), whereas patients without anemia were more likely to have experienced an ST-elevation MI (Group B = 38.9% and group D = 43.2%).

**Table 1:** Baseline Characteristics of patients presenting to hospital with MI

	*Group A Diabetes Anemia n = 716*	*Group B Diabetes No Anemia n = 1894*	*Group C No Diabetes Anemia n = 869*	*Group D No Diabetes No Anemia n = 3987*
Age [years] (SD)	74.0 (11.1)^bb,c,dd^	67.8 (12.5)^aa,cc,dd^	76.1 (13.2)^a,bb,dd^	64.2 (13.9)^aa,bb,cc^
Male [%]	51.8^b,c,d^	58.0^a,d^	58.8^a,d^	67.7^a,b,c^
BMI [kg/m^2^] (SD)	28.2 (6.6)^bb,cc^	29.6 (6.2)^aa,cc,dd^	26.1 (5.6)^aa,bb,dd^	27.7 (5.1)^bb,cc^
Systolic BP [mmHg] (SD)	133 (34)^bb,dd^	146 (33))^aa,cc,dd^	133 (32)^bb,dd^	142 (29)^aa,bb,cc^
Diastolic BP [mmHg] (SD)	75 (17)^bb,dd^	85 (18))^aa,cc^	75 (18)^bb,dd^	84 (21)^aa,cc^
Glucose [mg/dL] (mmol/L)	252 (14.0)^cc,dd^	256 (14.2)^cc,dd^	130 (7.2)^aa,bb^	130 (7.2)^aa,bb^
Hemoglobin [g/dL]	10.83^bb,cc,dd^	14.44^aa,cc^	11.10^aa,bb,dd^	14.50^aa,cc^
Total Chol [g/dL] (mmol/L)	173 (4.47)^bb,dd^	191 (4.95)^aa,cc,dd^	174 (4.50)^bb,dd^	198 (5.11)^aa,bb,cc^
LDL [mmol/L](mg/dL)	103 (2.66)^d^	114 (2.96)	109 (2.82)	128 (3.30)^a^
HDL [mmol/L] (mg/dL)	41 (1.05)	52 (1.34)	41 (1.07)	49 (1.28)
Creatinine [μmol/L] (mg/dL)	2.0 (180)^bb,cc,dd^	1.2 (110)^aa,cc,dd^	1.7 (153)^aa,bb,dd^	1.1 (100)^aa,bb,cc^
Creatinine Clearance [mL/min]	39.2^bb,dd^	61.4^aa,cc,dd^	41.5^bb,dd^	64.6^aa,bb,cc^
Creatinine Kinase [U/L]	835 ^bb,dd^	1072^aa,cc^	842^bb,dd^	1137^aa,cc^
CK-MB [U/L]	67^bb,dd^	61.4^aa,cc,dd^	41.5^bb,dd^	64.6^aa,bb,cc^
ST-elevation on ECG (%)	27.3^bb,c,dd^	38.9^aa,cc,d^	32.3^a,bb,dd^	43.2^aa,b,cc^

**a **signifies p < 0.05 between group A and current group. **b **signifies p < 0.05 group B and current group. **c **signifies p < 0.05 between group C and current group. **d **signifies p < 0.05 between group D and current group. Double symbol (e.g. **aa**) signifies p < 0.001.

### Cardiac risk factors

These are summarized in Table 2. Patients with anemia were less likely to have family history of premature coronary artery disease (group A 10.8%, group C 13.1%, group B 19.6%, and group D 32.2%). The incidence of previous MI (group A 32.0%, group B 26.1%, group C 27.3%, and group D 17.9%) and CHF (group A 27.9%, group B 12.0%, group C 18.8%, and group D 4.8%) was significantly higher in the presence of anemia, diabetes or both. More patients with DM had a previous history of hypertension (group A 63.3%, group B 61.4%, group C 51.4%, and group D 43.7%, all between group comparisons, p < 0.001, except A vs. B, p = ns)

**Table 2:** Cardiac Risk Factors

	**Group A Diabetes Anemia n = 716**	**Group B Diabetes No Anemia n = 1894**	**Group C No Diabetes Anemia n = 869**	**Group D No Diabetes No Anemia n = 3987**
Family History of CAD (%)	10.8^b,c^	19.6^a,c,d^	13.1^b,d^	32.2^a,b,c^
Smoker or ex-smoker (%)	56.0^b,d^	63.9^a,c,d^	59.0^b,d^	69.2^a,b,c^
Previous Diagnosis HTN (%)	63.3^cc,dd^	61.4^cc,dd^	51.4^aa,bb,dd^	43.7^aa,bb,cc^
Previous MI (%)	32.0^b,d^	26.1^a,d^	27.3^d^	17.9^a,b,c^
Previous CHF (%)	27.9^b,c,d^	12.0^a,c,d^	18.8^a,b,d^	4.8^a,b,c^

**a **signifies p < 0.05 between group A and current group. **b **signifies p < 0.05 group B and current group. **c **signifies p < 0.05 between group C and current group. **d **signifies p < 0.05 between group D and current group. Double symbol (e.g. **aa**) signifies p < 0.001.

### Outcome findings

All cause mortality was calculated separately as mortality within 30 days of admission, and mortality from 31 days to 36 months to reflect both short and long-term outcome. These data are summarized in Table 3. The short-term all-cause mortality in group A was 32.3%, group B was 16.1%, group C was 21.5%, and group D was 6.6% (all p < 0.001). The long-term mortality in group A was 47.6%, group B was 20.8%, group C was 34.3%, and group D was 10.4% (all p < 0.001). In terms of morbidity, there was no difference in the composite endpoint of admission for non-fatal re-infarction and/or CHF and/or or unstable angina between groups at 30 days. From 31 days to 36 months, group B had the highest number of events, which was significant when compared with the non-diabetic groups (group A 23.0%, group B 24.8%, group C 20.8%, and group D 22.1%, p < 0.05 for group B vs group C and D). When individual components of morbidity were studied between 31 days and 36 months, there were no significant differences in rates of admission for non-fatal reinfarction. CHF admissions were highest in patients with diabetes and anemia (group A 12.0%, group B 6.7%, group C 8.2%, group D 2.9%, all p < 0.001, except A vs C p < 0.05). For unstable angina, the patients with anemia had higher rates of readmission (group A 7.7%, group B 15.2%, group C 10.6%, group D 16.8%, all p < 0.001, except A vs. C and B vs. C, p < 0.05).

**Table 3:** Morbidity and Mortality

	**Group A Diabetes Anemia n = 716**	**Group B Diabetes No Anemia n = 1894**	**Group C No Diabetes Anemia n = 869**	**Group D No Diabetes No Anemia n = 3987**
**Mortality**				
≤30 days	231 (32.3)^bb,cc,dd^	305 (16.1)^aa,cc,dd^	308 (21.5)^aa,bb,dd^	263 (6.6)^aa,bb,cc^
31d – 36 months (% of 30d survivors)	231 (47.6)^bb,cc,dd^	331(20.8)^aa,cc,dd^	192 (34.3)^aa,bb,dd^	387 (10.4)^aa,bb,cc^
**Morbidity ≤30 days**				
Reinfarction	7 (1.0)	17 (0.9)	10 (1.2)	38 (1.0)
CHF admission	27 (3.8)^b,c,dd^	55 (2.9)^a,dd^	15 (1.7)^a^	57 (1.4)^aa,dd^
Unstable Angina (UA)	17 (2.4)^b,dd^	82 (4.3)^a,dd^	28 (3.2)^d^	226 (5.7)^aa,bb,c^
Composite end-point (Reinfarction, CHF, UA)	51 (7.1)	154 (8.1)	53 (6.1)	321 (8.1)
**Morbidity 31 days – 36 months**				
Reinfarction	24 (3.4)	55 (2.9)	18 (2.1)	97 (2.4)
CHF admission	86 (12.0)^bb,c,dd^	126 (6.7)^aa,cc,dd^	71 (8.2)^a,bb,dd^	116 (2.9)^aa,bb,cc^
Unstable Angina (UA)	55 (7.7)^bb,c,dd^	288 (15.2)^aa,c,dd^	92 (10.6)^a,b,dd^	668 (16.8)^aa,bb,cc^
Composite end-point (Reinfarction, CHF, UA)	165 (23.0)	469 (24.8)^c,d^	181 (20.8)^b^	881 (22.1)^b^

**a **signifies p < 0.05 between group A and current group. **b **signifies p < 0.05 group B and current group. **c **signifies p < 0.05 between group C and current group. **d **signifies p < 0.05 between group D and current group. Double symbol (e.g. **aa**) signifies p < 0.001. Percentages do not always add to total due to rounding.

**Figure 1:**
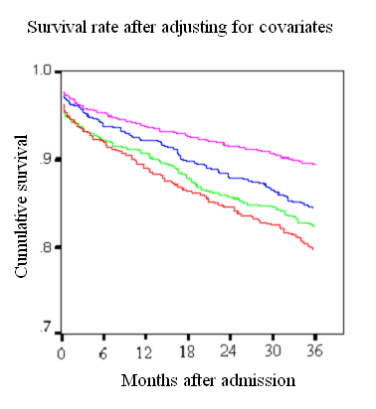
Survival rates after adjusting for covariates.

Table 4 summarizes the final step in each of the two regression models predicting mortality. Only variables having a significant relationship with the outcome, after controlling for the influence of the other covariates, were retained in the models. Diabetes was an independent risk factor in both short and long-term mortality (30-day OR 1.45 [CI = 1.20–1.76], 36-month OR 1.61 [CI = 1.41–1.85], both p <0.001). Short-term mortality was not directly influenced by anemia once other variables were included. However, anemia on admission remained independently associated with mortality in the long-term (OR 1.59, CI = 1.38 – 1.85). Figure 1 shows the long-term survival rate of patients according to their DM and anemia status after controlling for the covariates listed in Table 4.

**Table 4:** Adjusted Odds Ratios Using a Backward Elimination Model for Mortality

	Mortality ≤30 days			Mortality 31d – 36 m		
	Adjusted OR	p-value	95% CI	Adjusted OR	p-value	95% CI

**Comorbid conditions**						
Diabetes	1.45	<0.001	1.20 – 1.76	1.61	<0.001	1.41 – 1.85
Anemia				1.59	<0.001	1.38 – 1.85
Age (per year)	1.01	0.01	1.00 – 1.02	1.03	<0.001	1.03 – 1.04
Smoking				1.23	0.007	1.06 – 1.42
Previous MI				1.55	<0.001	1.34 – 1.79
**On Admission**						
STEMI	1.26	0.02	1.04 – 1.53			
Systolic BP >140 mmHg	0.68	<0.001	0.56 – 0.83			
High LDL	0.76	0.02	0.61 – 0.95			
Creatinine Clearance per ml/min	0.99	0.007	0.99 – 1.00	0.98	<0.001	0.98 – 0.99
**Therapy during hospitalization**						
CABG	0.59	0.02	0.39 – 0.89	0.58	<0.001	0.46 – 0.72
PCI				0.52	<0.001	0.41 – 0.66
Thrombolysis				0.58	<0.001	0.48 – 0.70
Medication on discharge						
B-blocker	N/A			0.64	<0.001	0.54 – 0.74
ASA	N/A			0.70	<0.001	0.60 – 0.82
Statin	N/A			0.79	0.002	0.68 – 0.91

**Figure 2a:**
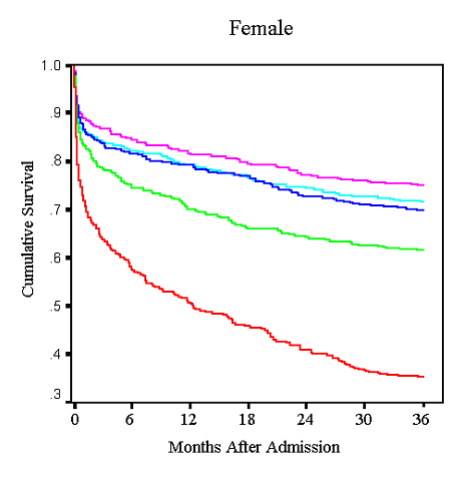
Unadjusted female survival by hemoglobin quintile.

**Figure 3:**
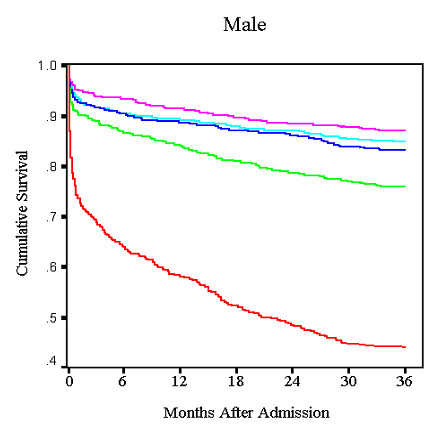
Unadjusted male Survival by hemoglobin quintile.

The overall mortality in each group containing patients with anemia was higher than in the corresponding non-anemic groups. Similarly, there was a drop in survival to 36 months concomitant with a low hemoglobin level at admission (Fig 2 and 3).

### Cause of death

The cause-specific mortality data are summarized in table 5. At 30 days, the overwhelming majority of patient deaths were due to cardiovascular disease (group A 73.2%, group B 82.6%, group C 73.8%, and group D 85.9%). By 36 months, cardiovascular death remained the most common cause of mortality overall (group A 43.7%, group B 54.1%, group C 47.0%, and group D 50.8%, group A to B p < 0.05) but between 50% and 60% of deaths were now due to competing etiologies across groups. Patients in group C had a higher rate of death due to cancer at 30 days (7.0% vs group A 1.7%, group B 0.0%, group D 0.8%, all p < 0.05). However, at 36 months, this difference lost some significance. Although there was a higher 30-day mortality due to gastrointestinal causes in the two anemia groups, (group A 3.5% and group C 2.7% vs. groups B 0.3% and D 0.8%) no difference in mortality due to gastrointestinal disease was seen at 36 months.

**Table 5:** Mortality at 30 Days and 36 Months by Cause of Death

**Cause of death within 30 days**	**Group A Diabetes Anemia (% deaths <=30d)**	**Group B Diabetes No anemic (% deaths <=30d)**	**Group C No diabetes Anemia (% deaths <=30d)**	**Group D No diabetes No anemia (% deaths <=30d)**
Cancer	4 (1.7)^c^	0 (0.0)b	13 (7.0)^a,dd^	2 (0.8)^cc^
Cardio	169 (73.2)^b,dd^	252 (82.6)^a,c^	138 (73.8)^b,d^	226 (85.9)^aa,c^
Stroke	3 (1.3)	10 (3.3)	4 (2.1)	6 (2.3)
Diabetes	18 (7.8)^cc,dd^	17 (7.4)^cc,dd^	0 (0.0)	0 (0.0)
G I cause	8 (3.5)^bb^	1 (0.3)^aa,cc^	5 (2.7)^bb^	2 (0.8)
Infection	5 (2.2)	8 (2.6)	3 (1.6)	9 (3.4)
Renal	4 (1.7)	3 (1.0)	5 (2.7)	2 (0.8)
Lung Disease	3 (1.3)	3 (1.0)	6 (3.2)	7 (2.7)
Other	16 (6.9)	11 (3.6)	12 (6.4)	8 (3.0)
Unknown	1 (0.4)	0 (0.0)	1 (0.5)	1 (0.4)
Total	**231**	**305**	**187**	**263**

**Cause of death 31 days to 36 months**	(% deaths 30 days – 36 months)			

Cancer	29 (12.6)	32 (9.7)^c^	42 (17.9)^b^	56 (14.5)
Cardio	101 (43.7)^b^	179 (54.1)^a^	110 (47.0)	196 (50.8)
Stroke	5 (2.2)	18 (5.4)	13 (5.6)	23 (6.0)
Diabetes	34 (14.7)^cc,dd^	36 (10.9)^cc,dd^	1 (0.4)^aa,bb^	3 (0.8)^aa,bb^
G I cause	10 (4.3)	8 (2.4)	7 (3.0)	9 (2.3)
Infection	12 (5.2)^bb^	11 (3.3)^aa^	10 (4.3)	11 (2.8)
Renal	10(4.3)	4 (1.2)	10 (4.3)	10 (2.6)
Lung Disease	9 (3.9)	17 (5.1)	9 (3.8)	25 (6.5)
Other	13 (5.6)^c^	18 (5.4)^c,d^	29 (12.4)^a,b^	38 (9.8)^b^
Unknown	8 (3.5)	8 (2.4)	3 (1.3)	15 (3.9)
Total	**231**	**331**	**234**	**386**

**a **signifies p < 0.05 between group A and current group. **b **signifies p < 0.05 group B and current group. **c **signifies p < 0.05 between group C and current group. **d **signifies p < 0.05 between group D and current group. Double symbol (e.g. **aa**) signifies p < 0.001.

## Discussion

The current study extends previous knowledge about both the short and long term risk of death in patients who present with an AMI. The purpose of this study was two-fold. First, we wanted to assess the impact of anemia and DM together on outcomes of patients with AMI. Second, we sought to identify cause-specific mortality in patients with anemia. Though our results confirm anemia as an independent risk factor for mortality in the post-MI population, they differ significantly from other published data[1,2,12]. While recent reports have shown that anemia is an independent predictor for short-term mortality[1,2], our data suggest that low hemoglobin is a risk factor for long-term mortality only. One possible explanation for this discordance may be the differences in both study design and the definition of anemia. For instance, a previous study of elderly medicare patients reported a negative correlation between 30-day mortality and progressively lower hematocrit levels, but this was unadjusted mortality[12]. Similarly, Lipšic et al. found higher adjusted 30-day mortality when a hemoglobin of 100 g/L was used as criteria for anemia, but did not correct for as many potential confounders as we did[2]. Furthermore, Sabatine et al., in a post-hoc analysis of the TIMI trials, did not find an increase in cardiovascular mortality, myocardial infarction, or recurrent ischemia in NSTEMI patients until hemoglobin values dropped below 110 g/L[1]. In our study, we defined anemia as a hemoglobin <120 g/L in females and <130 g/L in males. When analyzed by quintile, the impact of anemia on survival was more pronounced in the lowest quintile of hemoglobin for both men and women regardless of their DM status. Our study may have not shown a difference in short-term mortality because it examined any degree of anemia. Milder cases of anemia may have had minimal impact on short-term outcome and could therefore have masked any potential mortality risk in patients with more severe anemia.

Another possible explanation for this disparity may have to do with the differences in transfusion rates among groups. A recent report suggests a potential detrimental effect of blood transfusion in acute coronary syndrome[13]. We did not have any information about the frequency of blood transfusion in these patients.

In our study, mortality at 36-months was significantly increased in both anemia groups. The combination of diabetes and anemia was associated with a significant risk of death over 36 months, with almost 65% of patients dying by 36 months. The reasons for the increased long-term mortality may be multifactorial. Anemia has been shown to be well tolerated in the normal functioning heart[14]. The increase in cardiac output via a higher heart rate, larger stroke volume, and decrease in blood viscosity compensates for the decreased oxygen carrying capacity of the blood. [15-17] These compensatory mechanisms are mediated partially through activation of the sympathetic nervous system, which has been shown to be deleterious in coronary artery disease. [18-20] In animal studies, anemia in the presence of a fixed coronary artery stenosis reduces the ability of the heart to increase cardiac output and thereby results in left ventricle dysfunction[21,22]. Exacerbation of left ventricular dysfunction or ischemia by anemia in the post-MI patient may be partially responsible for the excess long-term mortality in this population.

Anemia could result from a variety of diseases, which may have an impact on the overall survival. Therefore, to address this issue we also examined cause-specific mortality. In our study, the majority of patients in both anemia and non-anemia groups died of cardiovascular disease both within 30 days and at 36 months. The rate of cardiovascular death was only different among patients with diabetes, with and without anemia. Unfortunately, a significant number of patients in the DM groups (14.7% with anemia and 10.9% without anemia) had their cause of death listed as 'diabetes'. This may have misclassified many deaths as non-cardiac, when in fact they may well have died from cardiovascular disease. More importantly, patients with anemia did not have significantly higher rates of death from non-cardiac causes such as cancer or hemorrhage, indicating that not only were mortality rates higher in both anemia groups, but the majority of people still died of cardiovascular disease.

We found no between group difference in the composite morbidity endpoint at 30 days, which may have been related to the high mortality among patients with both DM and anemia. Clearly, patients who died during their index hospitalization could not have been expected to have later events. When we excluded patients dying within 30 days, there was a trend towards more readmissions for CHF, unstable angina, and recurrent nonfatal MI in the DM and anemia group. CHF admissions and unstable angina admissions tended to be inversely related, although the only group with a higher incidence of readmission for CHF than unstable angina was that with both DM and anemia. Silent ischemia and CHF are certainly well recognized complications of DM and anemia respectively. [23-28]

This is a retrospective observational study with all the inherent limitations of such a design. Unrecorded comorbidities, treatment decisions, or interventions such as blood transfusions may have influenced mortality rates within the four groups, although we corrected for an extensive list of potential confounders with the exception of transfusions. We could only identify readmissions for those conditions listed and other outcome differences may have existed between study cohorts. However, CHF, unstable angina and nonfatal recurrent MI are the most common reasons for readmission following AMI. We were not able to identify cause of death in 1.7% of patients, although classification issues in such a small percentage of all study patients are unlikely to have systematically biased our results in any direction. By contrast, conditions that are not generally understood to lead directly to death (e.g. diabetes and hypercholesterolemia) were listed as a primary cause of death in a measurable number of patients. If anything, this would only be expected to strengthen the association between anemia and cardiac disease, as these conditions typically lead to death through their contributions to cardiovascular disease. Although the accuracy of coding of the Vital Statistics Registry of the province of Nova Scotia has not been published, the Canadian registries, when compared to other vital statistics registries, have been reported to have 100% completeness, and a low rate of coding to ill-defined causes of death (7%)[29].

Strengths of our study include the fact that data were obtained on a large and contemporaneous, population-based cohort of consecutively hospitalized patients across an entire health care system. Furthermore, the data collected were rich in clinical detail. Finally, we had access not only to all-cause mortality but also on cause-specific death courtesy of our ability to link to the provincial Vital Statistics Registry.

## Conclusion

In a large consecutive and unselected population of patients hospitalized with an AMI, we have confirmed diabetes as being a strong independent risk factor for both short and long-term mortality. The results of our study suggest that anemia, when considered as a dichotomous variable by analogy to diabetes, is not an independent risk factor for 30-day mortality; however, it is associated with reduced survival over the longer term and, moreover, the greater the degree of anemia the higher the mortality rate out to 36 months. To our knowledge, this is the first study showing that any degree of anemia has an independent adverse effect on long-term mortality post-myocardial infarction. The combination of diabetes and anemia is particularly unfavorable as 65% of patients in this group had died at 36 months. We have also found that the primary cause of death in patients with anemia is cardiovascular disease and not other conditions such as cancer, hemorrhage, or renal failure. Further research is now needed to examine whether correction of anemia is a means of reducing long-term mortality in affected patients hospitalized with an AMI.

## Competing interests

The ICONS registry was established through an unrestricted educational grant from Merck Frosst Canada and Co. while ongoing data acquisition and project management is funded by the Nova Scotia Department of Health. These sponsors had no role in the design, analysis, interpretation, review, or approval of the manuscript.

## Authors' contributions

DS, TR, and SI were involved in conception, design, interpretation of data associated with the study, and draft versions of the manuscript. SG, RR, and SK were involved in writing the introduction and revision of the manuscript. CO was involved in the analysis of the data, development of the figures, and wrote the methods section of the manuscript. JC and EU were involved in interpretation of the data as well as revisions of the manuscript for content. All authors read and approved the final manuscript.
